# Shaping the spin wave spectra of planar 1D magnonic crystals by the geometrical constraints

**DOI:** 10.1038/s41598-022-24969-x

**Published:** 2022-11-30

**Authors:** Justyna Rychły-Gruszecka, Jakob Walowski, Christian Denker, Tobias Tubandt, Markus Münzenberg, Jarosław W. Kłos

**Affiliations:** 1 Institute of Molecular Physics, Polish Academy of Sciences, Mariana Smoluchowskiego 17, 60-179 Poznan, Poland; 2 ISQI, Faculty of Physics, Adam Mickiewicz University Poznań, Uniwersytetu Poznańskiego 2, 61-614 Poznan, Poland; 3 Institut für Physik, Universität Greifswald, Felix-Hausdorff-Straße 6, 17489 Greifswald, Germany

**Keywords:** Ferromagnetism, Magnetic properties and materials, Spintronics, Magnetic devices

## Abstract

We present experimental and numerical studies demonstrating the influence of geometrical parameters on the fundamental spin-wave mode in planar 1D magnonic crystals. The investigated magnonic crystals consist of flat stripes separated by air gaps. The adjustment of geometrical parameters allows tailoring of the spin-wave frequencies. The width of stripes and the width of gaps between them affect spin-wave frequencies in two ways. First, directly by geometrical constraints confining the spin waves inside the stripes. Second, indirectly by spin-wave pinning, freeing the spin waves to a different extent on the edges of stripes. Experimentally, the fundamental spin-wave mode frequencies are measured using an all-optical pump-probe time-resolved magneto-optical Kerr-effect setup. Our studies address the problem of spin-wave confinement and spin-wave dipolar pinning in an array of coupled stripes. We show that the frequency of fundamental mode can be tuned to a large extent by adjusting the width of the stripes and the width of gaps between them.

## Introduction

Ferromagnetic stripes are one of the basic building blocks of magnonic structures^1–4^. They can act as waveguides carrying the information encoded in spin waves^5,6^. Recently, many publications have focused on the spin waves in the sets of stripes, arranged in-plane periodically (in magnonic crystals^7–10^) or quasiperiodically (in magnonic quasicrystals^11–15^). The stripe systems act not only as waveguides. By adjusting their dimensions, we can tailor the spin wave propagation and thus process magnonic signals^16,17^.

The collective magnetization dynamics in stripe arrays depend on the magnetic coupling between the stripes. Thus, the simplest possible structure of this type is a periodic sequence of flat stripes separated by air gaps, which can be carved in thin ferromagnetic films. In such a system, the coupling is provided by a dynamic stray field within the gaps. The strongest dynamic stray field is created by the fundamental mode, where the magnetization precesses in phase. The largest surface magnetic charges and, thus, the strongest dynamic stray field appears on the lateral faces between the stripes, which is also reflected in the ellipticity of precession^18^. However, this effect is partially mitigated by the reduction of precession amplitude close to the lateral faces for flat stripes, which is called dynamical pining of magnetization^19,20^. In other words, the system partially trades the demagnetizing energy related to the presence of surface charges on lateral faces (and related to the presence of dynamic stray field) for other contributions of demagnetizing energy, resulting from the volume magnetic charges induced by the non-homogeneous distribution of the spin-wave amplitude^21^. The pinning partially confines the fundamental mode within the stripes and causes a frequency up-shift when the stripe’s widths are narrowed. Another effect is the weakening of dipolar coupling between the stripes due to dynamic magnetization pinning because of the reduction of dynamic stray field.

The strength of the pinning is expressed by the pinning parameter^20^: $\left. \partial _{{\textbf{n}}} {\textbf{m}}({\textbf{r}})/{\textbf{m}}({\textbf{r}})\right| _{{\textbf{r}}={\textbf{r}}_0}$ – the ratio of the directional derivative of dynamic magnetization $\partial _{\textbf{n}} {\textbf{m}}({\textbf{r}})$ (in the direction normal to the edges ${\textbf{n}}$) to the magnetization amplitude itself ${\textbf{m}}({\textbf{r}})$, taken on the edge of the stripe ${\textbf{r}}={\textbf{r}}_0$. The pinning parameter varies from 0 to $\infty$, corresponding to the completely free ($\partial _{\textbf{n}}{\textbf{m}}({\textbf{r}})=0$) and pinned magnetization ($\left. {\textbf{m}}({\textbf{r}})\right| _{{\textbf{r}}={\textbf{r}}_0}=0$).

In the absence of the dipolar interaction, the spin wave is free on the surfaces ($\partial _{\textbf{n}}{\textbf{m}}({\textbf{r}})=0$)^22^. Then, the pinning can be introduced by a different mechanism: the presence of surface magneto-crystalline anisotropy^22^. In the general case, the dipolar pinning can be combined with the influence of surface anisotropy^20^. However, for thin stripes (where the thickness and width are tens and hundreds of nanometers, respectively), the dipolar interactions significantly impact pinning^20^.

When the dipolar interactions prevail over the exchange interactions, spin wave modes in confined geometry (e.g., in stripes) can be calculated by solving the integral eigenvalue problem with the magnetostatic Green’s function as an integral kernel^20,23–25^. This approach can be applied to flat stripes^19^ or arrays of flat stripes^26^. The underlying theoretical model^26^ was successfully confirmed by experimental studies where the spin-wave spectra in stripe arrays were measured using Brillouin Light spectrometry^27–29^. It is worth noting that the spin-wave dynamics in a sequence of flat stripes can also be successfully described in the lattice (Hamiltonian-based) models with dipolar and exchange interactions taken into account^30,31^.

Here, we investigate experimentally and theoretically the effect of dipolar interaction between magnetic nanoelements (stripes) on spin wave pinning and consequently on the frequency of the fundamental mode in such structures. Spin wave dynamics is calculated numerically by solving the linearized Landau-Lifshitz equations. We use an experimental technique complementary to Brillouin Light Spectrometry^29,32,33^, the time-resolved magneto-optical Kerr effect (TR-MOKE) spectroscopy^34–37^, to determine the fundamental mode frequency. Using the TR-MOKE technique, we measure the frequency of the fundamental spin-wave mode (precisely at the wave vector $k=0$), for which the dynamic dipolar field between nanoelements in planar structures is the strongest. We investigate the continuous transition from a periodic sequence of isolated stripes (separated by large air gaps) to a continuous layer (where the gaps vanish) to study the relation between the dipolar coupling in an array of stripes and the spin wave pinning on the edges of stripes.

The paper is organized in the following way. In “Results and discussion”, we present and describe the results of our measurements and computations. We show the dependence of frequency and mode profile of fundamental mode (i.e., the mode which precesses uniformly in-phase in the whole volume of the system) on the structure’s geometry—stripes width and size of the air gap. “Results and discussion” is finalized with the conclusions. The following section (“Methods”) describes the experimental methods (fabrication of the samples, TR-MOKE method), numerical techniques (finite element method (FEM)^38^), and theoretical approach (semi-analytical method based on magnetostatic Green’s function technique^23^) we used. We also provide the Supplementary Information, which describes: the details of post-processing for the TR-MOKE signal, the semi-analytical calculations of the demagnetizing factors for fundamental mode, and the determination of equilibrium orientation of static magnetization.

## Results and discussion

**Figure 1:**
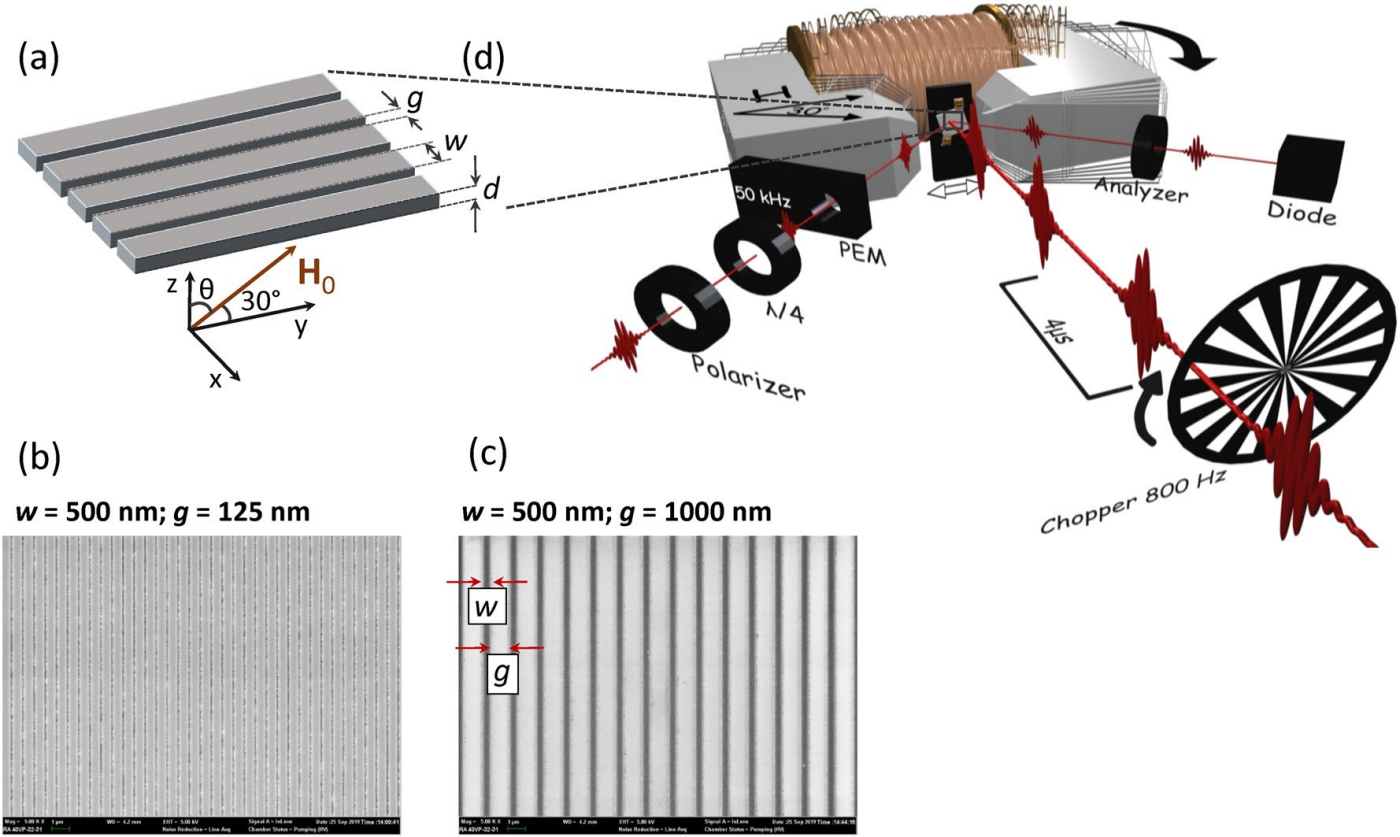
The geometry of the investigated system. (**a**) The periodically ordered CoFeB stripes have a cross-section of thickness $d$ and width $w$, and are separated by air gaps of width $g$. The vector ${\textbf{H}}_{\mathrm{{0}}}$ shows the applied magnetic field direction. (**b**) The EBL scan of the structure with $w=500\,\text{nm}$ and $g=125\,\text{nm}$. (**c**) The electron-beam lithography (EBL) scan of the structure with $w=500\,\text{nm}$ and $g=1000\,\text{nm}$. The stripes are deposited on $\mathrm {Si0_2}$ substrate. (**d**) The scheme of the all-optical pump-probe TR-MOKE setup.

The systems consist of CoFeB stripes of finite width $w$ and thickness $d$. The stripes are periodically aligned in-plane and separated by air gaps $g$—see Fig. 1a. We are interested in the planar system where the in-plane dimensions (widths of the stripes $w$) are much larger than the thickness $d$: $w\gg d$. For such geometry, we can only observe the spin wave pinning on the lateral faces of the stripes and relate the spin-wave confinement to the finite width of the stripes $w$ and the interaction between the stripes (determined by the width of the air gaps $g$).

In our studies, we fixed the thickness $d =$ 30 nm and varied the width $w$ of the stripes (in the range: 500–1000 nm) and the air gaps $g$ between them (in the range: 125–1000 nm). The system is magnetized by a uniform in-plane bias magnetic field of magnitude $\mu _{0}H_{0}$ = 0.15 T tilted at an angle $\theta =60^{\circ }$ out of the normal to the plane ($z$-axis), in the direction of the stripes ($y$-axis). In all calculations, we have assumed the saturation magnetization $M_{\mathrm{{S}}} =$ 1342 kA/m and the exchange constant $A_{\mathrm{{exch}}}~=$ 13.6 pJ/m. The gyromagnetic ratio is $\gamma =-176$ rad GHz/T. Those values come both from measured values of uniform CoFeB film, as well as from matching the frequency value for a homogeneous CoFeB layer in numerical calculations with the value obtained in measurements with TR-MOKE (the result for a uniform layer is presented in Supplementary information A). The same fundamental mode frequency could be obtained for a uniform layer using Kittel’s ferromagnetic resonance (FMR) frequency formula: $f=\frac{\gamma \mu _{0}}{2\pi }\sqrt{(H_{0}\sin \theta )(H_{0}\sin \theta +M_{\mathrm{{S}}})}$. The TR-MOKE experiment was performed as presented in Fig. 1d and described in “Methods-measurements”. The signal measured in time was then subjected to postprocessing (described in Supplementary information A), culminating in the calculation of the Fourier transform of the measured and processed signal. With this procedure, we are able to obtain the experimentally measured frequencies for each of the fabricated structures.

**Figure 2:**
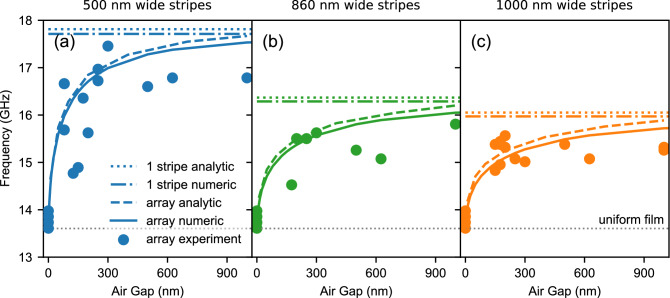
The results show the fundamental mode frequency for individual structures, depending on the width of the air gap (separation) between magnetic stripes with the widths of (**a**) 500 nm, (**b**) 860 nm, and (**c**) 1000 nm. In subplots (**a**), (**b**), and (**c**), the results of measurements with the TR-MOKE method are represented by points. These results are compared with the solid and dashed lines’ numerical and semi-analytical calculations. The dotted grey line shows the lower frequency limit corresponding to the fundamental mode frequency of the layer (the result of the calculation). The dotted (dash-dotted) lines show the frequency limits for individual stripes of given widths, which is the upper-frequency limit of the tested systems—sequences of stripes of a given stripe width—the result of numerical (semi-analytical) calculations.

The numerically calculated FMR frequency for the CoFeB layer is equal to 13.6 GHz. The FMR frequencies obtained experimentally by TR-MOKE are in the range of 13.6 up to 13.95 GHz. Differences may be due to the quality of the samples measured, the quality of the laser in the pump-probe experiment, and other factors. It follows that measurement uncertainties of half a gigahertz are expected, even for simple structures. The value calculated for uniform CoFeB film is marked in Fig. 2 by a dotted grey line.

After these preliminary studies, we performed (by TR-MOKE) the measurements of magnetization dynamics in all the fabricated periodic structures with stripes of the widths $w =$ 500, 860, 1000 nm, separated by the air gaps with the widths $g =$ 125, 150, 175, 200, 250, 300, 500, 625, 1000 nm. To demonstrate the impact of the width of the stripes on the interactions between the neighboring stripes in periodic structures, we have plotted the frequency of the fundamental mode in the dependence on the air gaps’ width $g$ (dots in Fig. 2). These dependencies were shown in separate sub-figures, for each considered width of the stripes $w$. To verify the measurement, we added the results of numerical simulations and semi-analytical calculations, which are presented by solid and dashed lines, respectively. The numerical studies were done in COMSOL Multiphysics and based on FEM simulations (see “Methods B”), whereas the formalism of tensorial magnetostatic Green functions was used for semi-analytical calculations (see Supplementary information B).

It can be seen that we have achieved good agreement between experimental, computational, and semi-analytical results. Experimental results are subject to significant measurement error even for uniform CoFeB films due to the quality of the CoFeB material (homogeneity of composition), measurement uncertainty (such as laser settings, and measurement of structures in the right place), which we have already described for the layers. In the case of periodic structures, the experimental measurement results are subject to even more significant uncertainty due to the difficulty of producing ideal thin magnetic stripes with sharp edges, separated by air gaps of the same width along the length of the stripe. Taking this into account, the presented experimental results are within the measurement uncertainty limit. It can be seen that the fundamental mode frequencies depend on the air gap separations between stripes. The change of fundamental mode frequency between uniform film and periodic structures is continuous. For very small separations, the fundamental mode frequency of periodic structure tends to be the FMR frequency of a uniform film, being the bottom limit. The bigger the separations, the higher the frequency of the fundamental mode of the structure. The fundamental mode frequency is the highest for completely separated stripes—i.e., single stripes—which is the upper limit. In our measurements, it is impossible to measure a signal from a single stripe since the signal would be too small, so the upper limit was only calculated numerically (dash-dotted line) and semi-analytically (dotted line). The fundamental mode frequency of a single stripe is presented as a dotted (dash-dotted) line for each subfigure in Fig. 2 independently.

We can notice (in Fig. 2) that the measured frequencies (points) are mostly lowered compared to the corresponding numerical and semi-analytical results (solid and dashed lines). This is due to the fact that the ferromagnetic stripes obtained in the fabrication process are, in most cases, slightly wider than those planned in the design. In general, the smaller the structures, the lower the accuracy of their fabrication (the narrower the stripes, the higher the uncertainty associated with their fabrication, and the narrower the air gaps between the stripes, the worse they are reproduced up to the limit of the production possibilities of a given apparatus. The upper-frequency limit of the structures (obtained for single stripes) varies depending on the geometry of the stripe, in our case, its width. It is worth noting that the TR-MOKE signal is weaker for wide gaps due to the smaller amount of magnetic material than in other samples. This causes the accuracy of frequency determination for the fundamental mode to be limited. The reason is that due to the damping of precessing magnetization, a sufficiently long signal above the noise level cannot be recorded. The effect of stripe width on the fundamental mode frequency is easily read from Fig. 2 by comparing the dotted lines—the narrower the stripe, the higher the fundamental mode frequency.

**Figure 3:**
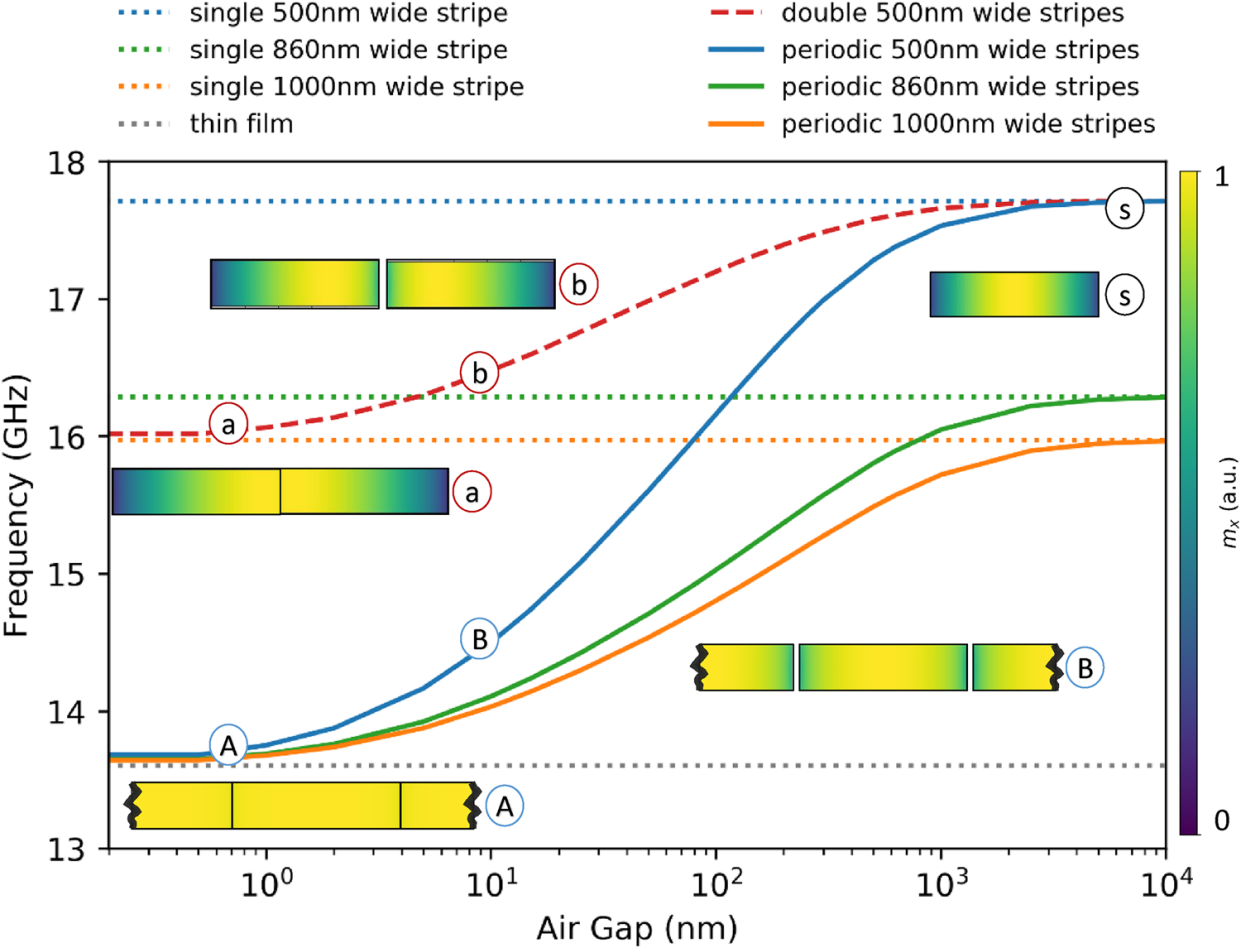
The results show the fundamental mode frequency for individual periodic structures, depending on the width of the air gap (separation) between the magnetic stripes with the widths of 500 nm (blue line), 860 nm (green line), and 1000 nm (orange line), calculated numerically using FEM. The dotted lines present fundamental mode frequencies of the limiting structures - uniform thin CoFeB film, which is the limiting case of the periodic structure of vanishing separation between stripes (grey dotted line), and limits of single stripes, which is the case when stripes are as far that they cannot feel each other any longer—separated dotted lines are presented for each individual width of stripe—$w =$ 500 nm (blue dotted line), $w =$ 860 nm (green dotted line), $w =$ 1000 nm (orange dotted line). The red dashed line shows the FEM result for a structure consisting of only two stripes—the upper limit is a single 500 nm wide stripe, and the lower limit is a single 1000 nm wide stripe. Additionally, the spin wave mode profiles are presented for structures with $w =$ 500 nm: (A) periodic structure with $g =$ 0.5 nm; (B) periodic structure with $g =$ 10 nm; (a) double stripes with $g =$ 0.5 nm; (b) double stripes with $g =$ 10 nm; (s) single stripe.

For a better understanding and more explicit interpretation of the experimental results, we performed the simulations for the extended range of the widths of air gaps *g*, i.e., from $g=$ 0.5 nm up to $g=$ 10 $\upmu$m. This allows us to present the relation between the frequency of the fundamental mode and the width of the air gaps on the logarithmic scale in Fig. 3 and observe the transition between the continuous layer and the sequence of isolated stripes as the distance between the stripes increases—see the blue, green and orange curves for the widths of stripes: $w=$ 500, 860, 1000 nm. We also presented additional results for single pair of stripes of 500 nm width each, separated by an air gap, for which the dependence of the fundamental mode frequency on the air gap is indicated in Fig. 3 by the red dashed line. For double $w=$ 500 nm wide stripes, the upper limit for fundamental mode frequency is just the frequency for a single stripe of the width $w=$ 500 nm—as is the case for a periodic structure composed of such stripes, whereas the lower limit for fundamental mode frequency is the fundamental mode frequency of a single stripe of the width $w=$ 1000 nm. This is because of the merging of two $w=$ 500 nm stripes into one stripe of double width, for $g \rightarrow 0$. Similarly, the lower limit of the fundamental mode frequency for a periodic stripe structure is the FMR frequency of a layer.

We have plotted the profiles of the fundamental mode for systems composed of stripes of one selected width ($w=$ 500 nm) to illustrate the relation between the frequency of the fundamental mode and spin-wave pinning. The colors for the modes’ profiles denote the magnitude of the in-plane component of dynamic magnetization $m_x$. The dark and light colors correspond to the small and large precession amplitude, respectively. We can observe that the amplitude distribution of all modes is homogeneous through the stripes’ thickness, while across the width are partially pinned at the edges of the stripes, depending on the separation from other stripes.

The profile marked with the label (s) is the profile of the mode of a single 500 nm wide stripe (or the stripe separated from the other stripes by a considerable distance: $g\rightarrow \infty$). We can observe quite a strong pinning of magnetization dynamics at the edges of the stripe. The magnetization dynamics is more released at the stripe’s edges for the case of a $g=$ 10 nm stripe gap, denoted by label (B). In the limit of an extremely narrow separation between the stripes ($g=$0.5 nm, marked by (A)), it can be seen that the mode is practically homogeneous, and the magnetization is almost completely free at the edge of the magnetic stripe, almost entirely resembling the magnetization in the magnetic layer.

We performed the supplementary calculations for a single pair of stripes of the width $w=$ 500 nm each. The limit (s), denoting the fully separated stripes (for $g\rightarrow \infty$), is the same as the periodic structure, while the lower frequency limit (for $g\rightarrow 0$) and the mode profile are the same as for a $w=$ 1000 nm wide stripe. In the limit of very narrow separation between two stripes (0.5 nm, denoted by the label (a)), it can be seen that the spin-wave amplitude has a maximum value near the boundary between the two stripes, while at the external edges, the spin-wave is dipole-pinned, as is the case for the separated stripe. In the case of an intermediate separation between the stripes ($g=$ 10 nm, labeled (b)), the spin wave is partially released at the interface between the stripes while maintaining identical pinning at the outer edges of the stripes.

The pinning of the spin waves means the partial confinement inside the stripes, which lifts the frequencies of the spin wave. This effect is much more substantial for narrower stripes^19,21,27^. The increase of the frequencies for the wave eigenmodes caused by confinement is a general wave phenomenon that also manifests in considered magnonic structures when the spin wave pinning comes into play.

We can summarize our research on the magnetization dynamics in the array of flat stripes arrays by highlighting the following conclusions. (i) The fundamental mode frequency increases with the stripes’ separation. It also depends on the width of the stripe due to the pinning. The frequency of the fundamental mode is lower for wider stripes. This decrease results not only from the extension of the space on which the mode is spanned (i.e., the width of the stripe) but also from the reduction of pinning (when the ratio of width to thickness is increasing—as it was also shown in Ref.^19^). It is worth noting that the wider stripes are more strongly coupled compared to the narrower stripes, at the same gaps, because the weaker pinning means the stronger stray field produced at the lateral faces of the steps, which is responsible for coupling. (ii) The dipolar interactions between the constituting nanoelements of magnonic planar nanostructure must be considered even if the distance between successive stripes is within a single micrometer. (iii) The TR-MOKE measurements allow for the investigation of the frequency of fundamental mode, i.e., the mode precessing uniformly in space (with wave vector $k=0$), which are inaccessible for BLS measurements. However, the resolution of TR-MOKE measurements in the frequency domain is weaker, and the determination of the spin waves frequencies is less accurate. (iv) The FEM computations can be used to investigate the spin-wave dynamics in the frequency domain in considered systems. However, the semi-analytical calculations (based on the formalism of magnetostatic Green functions) provide sufficiently accurate results, and the assumptions made in this method are not problematic for the systems studied here.

## Methods

### Fabrication

The investigated $\text{CoFeB}$ stripe patterns are fabricated in two steps. First, the $30\,\text{nm}$ $\text{CoFeB}$ film is deposited on a $\mathrm {SiO_{2}}$ substrate by magnetron sputtering from a $\mathrm {Co_{40}Fe_{40}B_{20}}$ target using $\text{Ar}$ plasma at a base pressure in the order of $\sim \mathrm {10^{-8}}\,\text{bar}$ and the deposition rate set to $0.45\,\mathrm {nm/s}$. A $2\,\text{nm}$ $\text{Ru}$ capping is subsequently deposited in situ by e-beam evaporation to prevent surface oxidation. Afterward, patterns are written using a scanning electron microscope (SEM) into a $500\,\text{nm}$ thick photoresist layer. Following the developing process and Ar plasma etching into the ferromagnetic CoFeB layer, the resulting stripe patterns grouped in $200\,\mathrm {\mu m}\,\times \,200\,\mathrm {\mu m}$ squares are created (for more information, see Supplementary Material). The final patterns have the following dimensions, stripe widths $w=500\,\text{nm}$, $860\,\text{nm}$, and $1000\,\text{nm}$, with air gaps between the stripes for each *w* ranging from $g=0\,\text{nm}$ (continuous film) to $g=1000\,\text{nm}$, see Fig. 1b,c which shows sections of example structures. Both images are recorded with an SEM and show stripe sequences of the same widths *w* but for two different air gap widths *g*. The darker grey color corresponds to the magnetic stripes, while the lighter grey represents the air gaps.

### Measurements

Magnetization dynamics for precession frequency determination is measured in a time-resolved magneto-optical Kerr-effect setup (TR-MOKE) in an all-optical pump-probe scheme, depicted in Fig. 1d. The measurements are performed using a Ti:Sapphire laser at a central wavelength set to $800\,\text{nm}$ (Coherent Mira Seed)^39^. The pulses are amplified by a regenerative amplifier (RegA 9040 by Coherent) at a repetition rate of $250\,\text{kHz}$ and compressed to $40\,\text{fs}$ pulse duration. Before the experiment, a beam splitter divides the laser beam propagating from the laser system into two beams, an intense pump beam for excitation and a weak beam to probe the magnetization. The sample is mounted inside a magnetic field between two pole shoes of an electromagnet. The magnetic field is directed along the shape anisotropy axis but tilted by $60^{\circ }$ out of the normal to surface plane, as the sketches in Fig. 1a,d indicate. In this geometry, at field strengths, $\mu _{0}H_{0}=150\,\text{mT}$, the stripes magnetization is saturated and slightly ($2^{\circ }$–$3^{\circ }$) tilted out of the easy magnetization axis. For such a large difference between the equilibrium orientation of magnetization and the direction of the external field, the torque driving the magnetization precession (after partial demagnetization of the sample by pumping pulse) is significant^40^.

The dynamics is triggered via a broadband excitation by depositing energy from the pump beam laser pulses at fluences adjusted to $12.7\,\mathrm {mJ/cm^2}$ focused down to $100\,\mathrm {\mu m}$ in diameter at the sample surface. We probe the change in magnetization by the time-delayed probe beam on a timescale up to $1\,\text{ns}$ and the temporal resolution set to $2\,\text{ps}$. All TR-MOKE measurements are performed using a double modulation technique. The MOKE signal is modulated by a photo-elastic modulator PEM at 50 kHz, and the signal is recorded via a photodiode by the first lock-in amplifier. The time-resolved change in magnetization is measured by modulating the pump beam using a mechanical chopper at 800 Hz, periodically interrupting the pump pulses, and thus recording the signal change from the first lock-in amplifier using a second lock-in amplifier.

### Theoretical and numerical framework

The magnetization dynamics in a magnetic system is described by the equation of motion of the magnetization, called the Landau–Lifshitz equation^18^:

1 $$\begin{aligned} \frac{\partial {\textbf{M}}({\textbf{r}},t)}{\partial t}= -|\gamma | \mu _0 \left[ {\textbf{M}}({\textbf{r}},t)\times {\textbf{H}}_{\text{eff}}({\textbf{r}},t) +\frac{\alpha }{M_{\mathrm{{S}}}} {\textbf{M}}({\textbf{r}},t) \times ({\textbf{M}}({\textbf{r}},t)\times {\textbf{H}}_{\text{eff}}({\textbf{r}},t) ) \right] , \end{aligned}$$

where $\gamma$ is the gyromagnetic ratio, $\mu _{0}$ is vacuum permeability, $\alpha$ is dimensionless damping coefficient, $M_{\mathrm{{S}}}$ is saturation magnetization. This differential equation describes the precessional motion of magnetization in a solid and is commonly used in micromagnetics to model the effects of a magnetic field on ferromagnetic materials. The first term in LLE (1) describes the precessional motion of magnetization around the direction of the effective magnetic field, and the second term enriches that precession with damping by dragging the magnetization vector towards the direction of the effective field^41^. Both the magnetization of the ferromagnet ${\textbf{M}}$ and the effective magnetic field depend on time and space; the latter can comprise many terms: ${\textbf{H}}_{\text{eff}}({\textbf{r}},t) = {\textbf{H}}_{\mathrm{{0}}}({\textbf{r}})+{\textbf{H}}_{\mathrm{{ex}}}({\textbf{r}},t)+{\textbf{H}}_{\mathrm{{d}}}({\textbf{r}},t)$. Those terms are: static, uniform external magnetic field ${\textbf{H}}_0$, long-range dipolar (magnetostatic) field ${\textbf{H}}_{\mathrm{{d}}}$, and short-range isotropic exchange field ${\textbf{H}}_{\mathrm{{ex}}}$. The exchange field could be expressed in the linear approximation as in^42^:

2 $$\begin{aligned} {\textbf{H}}_{\text{ex}}({\textbf{r}},t)= \nabla \cdot \left( \lambda _{\text{ex}}^2(x)\nabla {\textbf{M}}({\textbf{r}},t) \right) , \end{aligned}$$

where $\lambda _{\mathrm{{ex}}} = \sqrt{\frac{2A_{\mathrm{{ex}}}({\textbf{r}})}{\mu _0 M^{2}_{\mathrm{{S}}}({\textbf{r}})}}$ is the exchange length, and $A_{\mathrm{{ex}}}({\textbf{r}})$ is the exchange stiffness constant.

Dipolar field ${\textbf{H}}_{\mathrm{{d}}}$ can be derived from the magnetostatic Maxwell equations (that is, in the absence of electric currents and by neglecting the temporal changes of the electric vector):

3a $$\begin{aligned}&\mathbf {\nabla }\cdot ({\textbf{M}} + {\textbf{H}}_\text{d})= 0 , \end{aligned}$$

3b $$\begin{aligned}  \boldsymbol{\nabla } \times {\textbf{H}}_\text{d}=0. \end{aligned}$$

For the above conditions, it is possible to formulate the expression of the magnetostatic field such as:

4 $$\begin{aligned} {\textbf{H}}_{\text{d}} = - (\nabla \varphi ), \end{aligned}$$

where scalar potential $\varphi$ is called magnetostatic potential.

To observe the TR-MOKE signal, we applied the external field ${\textbf{H}}_0$ in the oblique direction (see Fig. 1a,d), with two components $H_0\cos \theta$ and $H_0\sin \theta$ oriented in the normal direction to the plane of stripes ($z-$direction) and along the stripes ($y-$direction), respectively. Due to the small value of the field $H_0$, compared to strong shape anisotropy (resulting from a large value of $M_{\text{S}}$ for CoFeB), the static magnetization is practically oriented along the stripes, with a very small (ca. $2.5^{\circ }$) out-of-plane deviation (see Supplementary information B for a detailed explanation). The linearization procedure, that we used, neglects the out-of-plane component of an external field and splits the magnetization vector and magnetic field into static: $M_{S}\,\hat{{\textbf{y}}}$, $H_0\sin \theta \,\hat{{\textbf{y}}}$ and dynamic: (radio-frequency) components ${\textbf{m}}=[m_x,0,m_z]$, ${\textbf{h}}=[h_x,0,h_z]$. We can neglect all nonlinear terms in the dynamical components. Since $|{{\textbf{m}}}({{\textbf{r}}},t)|\ll |M_{z}(y)|$, we can also assume $M_{z}(y)\approx M_{\text {S}}(y)$, where $M_{\text {S}}(y)$ is the saturation magnetization dependent on the *y*-coordinate. We consider monochromatic SWs in a fundamental mode (${\textbf{k}}=0$) that mainly contributes to the recorded signal. The time and space dependence of the dynamic component of the magnetization and the dipolar field is assumed to have the forms ${\textbf{m}}({\textbf{r}},t)={\textbf{m}}(x,z) e^{i\omega t}$, and ${\textbf{h}}({\textbf{r}},t)={\textbf{h}}(x,z) e^{i\omega t}$, where $\omega$ is the spin waves angular frequency ($\omega = 2 \pi f$ where *f* is the frequency).

Lastly, coupling the Landau–Lifshitz equation with Gauss law for magnetism, we arrive at the following system of equations for the dynamic magnetization ${\textbf{m}}$ and magnetostatic potential $\varphi$:

5a $$\begin{aligned} \partial _x(m_x - \partial _x \varphi ) + \partial _z(m_z - \partial _z \varphi ) = 0, \end{aligned}$$

5b $$\begin{aligned}  \frac{H_\text{0}\sin \theta }{M_\text{S}} m_x - \frac{i \omega }{|\gamma | \mu _0 M_\text{S}} m_z + \partial _x \varphi - \nabla \cdot (\frac{\lambda _{\text{{ex}}}^2}{M_\text{S}} \nabla m_x) = 0, \end{aligned}$$

5c $$\begin{aligned}&\quad \frac{H_\text{0}\sin \theta }{M_\text{S}} m_z + \frac{i \omega }{|\gamma | \mu _0 M_\text{S}} m_x + \partial _z \varphi - \nabla \cdot (\frac{\lambda _{\mathrm{{ex}}}^2}{M_\text{S}} \nabla m_z) = 0. \end{aligned}$$

For the eigenvalue problem (5), we neglected damping because the correction of the eigenfrequencies due to the damping is quadratic in $\alpha$, which is extremely small for CoFeB, where $\alpha <0.01$.

The TR-MOKE measurements give the frequency of the fundamental mode. Therefore, we only search for the lowest frequency solution of the eigenproblem (5), which corresponds, in our system, to the mode being homogeneous in phase. To calculate the frequencies (eigenfrequencies) and SWs mode profiles (eigenmodes) in the considered periodic structures, we employed the frequency-domain finite element method solving the eigenproblem obtained from Eq. (5). We have used the COMSOL Multiphysics software^38^ to solve^8^ Eq. (5). This technique allows us to solve the problem directly in the frequency domain and avoid the postprocessing required for micromagnetic simulations which are performed in the time domain. At the edges of the computational domain (far from the ferromagnetic materials, i.e. above and below the planar structure, in z-direction), the Dirichlet boundary conditions, forcing the magnetostatic potential to vanish, are imposed. An elementary cell is created for the structures under investigation, which, in the case of periodic structures, has Bloch boundary conditions imposed at the edges. Here, we reduced the Bloch boundary conditions to periodic boundary conditions by assuming that the wave vector: $k_x = 0$. This ensures the in-phase precession in the successive stripes, characteristic for the fundamental mode. The Dirichlet boundary conditions for single and double stripes are implemented at the far distance from a ferromagnetic material in the *x*-direction. For the resulting elementary cell, we fit a finite element mesh. We performed calculations in the mathematics module of COMSOL Multiphysics software, where we were able to enter the equations and boundary conditions of interest. Similarly as in our previous work^21^, the dipolar pinning of spin waves results from a dynamical demagnetizing field, which is included in our calculations; therefore, it does not have to be forced at the boundaries of a system.

Semi-analytical calculations supplemented the numerical results. The fundamental mode frequency was calculated using the Kittel formula for the planar system with the uniaxial shape anisotropy: $N_y=0$, $N_z=1-N_x\gg N_x$. We assumed that magnetization is aligned in-plane along the stripes. This assumption is valid when $H_0\ll M_{\text{S}}$ (see Supplementary information A for explanations):

6 $$\begin{aligned} f=\frac{\gamma \mu _{0}}{2\pi }\sqrt{\big (H_{0}\sin \theta +M_{\mathrm{{S}}} (1-N_z)\big )\big (H_{0}\sin \theta +M_{\mathrm{{S}}}N_z\big )}. \end{aligned}$$

The demagnetizing factor $N_z$ for fundamental mode was calculated by solving the integral eigenvalue problem for tensorial magnetostatic Green’s function (see Supplementary information B for details). For these semi-analytical calculations, we neglected the exchange interaction and assumed that the profile of the fundamental mode is uniform in the normal direction (i.e., *z*-direction) for the flat stripes ($w\gg d$).

## Supplementary Information

Supplementary Information.

## Data Availability

The datasets used and analyzed during the current study available from the corresponding author on reasonable request.
