# Supplementary material for: Shaping the spin wave spectra of planar 1D magnonic crystals by the geometrical constraints
Source: Sci Rep. 2022 Nov 30;12:20678. doi: 10.1038/s41598-022-24969-x (PMC9712760; doi:10.1038/s41598-022-24969-x)
Supplement: Supplementary file 1 — Supplementary Information. [file 41598_2022_24969_MOESM1_ESM.pdf]

Shaping the spin wave spectra of planar 1D magnonic crystals by the geometrical constraints: Supplementary information

Justyna Rychły-Gruszecka[1,2], Jakob Walowski[3], Christian Denker[3], Tobias Tuband[3], Markus Münzenberg[3], and Jarosław W. Kłos[2,*]

[1]Institute of Molecular Physics, Polish Academy of Sciences, Mariana Smoluchowskiego 17, 60-179 Poznań, Poland
[2]ISQI, Faculty of Physics, Adam Mickiewicz University Poznań, Uniwersytetu Poznańskiego 2, 61-614 Poznań, Poland
[3]Institut für Physik, Universität Greifswald, Felix-Hausdorff-Straße 6, 17489 Greifswald, Germany
[*]klos@amu.edu.pl

ABSTRACT

We present experimental and numerical studies demonstrating the influence of geometrical parameters on the fundamental spin-wave mode in planar 1D magnonic crystals. The investigated magnonic crystals consist of flat stripes separated by air gaps. The adjustment of geometrical parameters allows tailoring of the spin-wave frequencies. The width of stripes and the width of gaps between them affect spin-wave frequencies in two ways. First, directly by geometrical constraints confining the spin waves inside the stripes. Second, indirectly by spin-wave pinning, freeing the spin waves to a different extent on the edges of stripes. Experimentally, the fundamental spin-wave mode frequencies are measured using an all-optical pump-probe time-resolved magneto-optical Kerr-effect setup. Our studies address the problem of spin-wave confinement and spin-wave dipolar pinning in an array of coupled stripes. We show that the frequency of fundamental mode can be tuned to a large extent by adjusting the width of the stripes and the width of gaps between them.

A. TR-MOKE signal processing

The experimental data obtained from TR-MOKE measurements in the setup presented in the manuscript in Fig. 1(d) enable extracting fundamental mode frequency for the investigated structures using the Fast Fourier Transform algorithm (FFT). For an optimal fundamental mode frequency extraction, the data is processed in two steps. First, a an electronic and phononic background underlying the precessional data is deducted, by fitting a double exponential function (see Fig. 1(a)):

$$y = A_1 e^{-xt_1} + A_2 e^{-xt_2} \tag{1}$$

the obtained parameters $A1$, t_1, $A2$, and t_2 represent the amplitudes and lifetimes of the additional reflection in the magnetic signal stemming from electron and phonon excitations. Subtracting this background, as shown by the green line in Fig. 1(a) from the measured signal minimizes the amplitudes of undesired frequencies. In a second step, the signal is autocorrelated. An additional zero padding allows for frequency resolution enhancement. The subsequent FFT returns a precisely determined fundamental mode frequency . For comparison, Fig. 1(b) shows the frequency spectrum obtained by applying FFT on raw signal data (orange) and the obtained from processed data (blue). The row data spectrum shows a and additional emerging peak at lower frequencies along with a poor frequency resolution. In contrast, the high-quality processed signal, enables to extract the fundamental mode frequency satisfactorily accurately from the peak maximum. For the example, the data shown in Fig. 1(b), yield an FMR = 13.85 GHz.

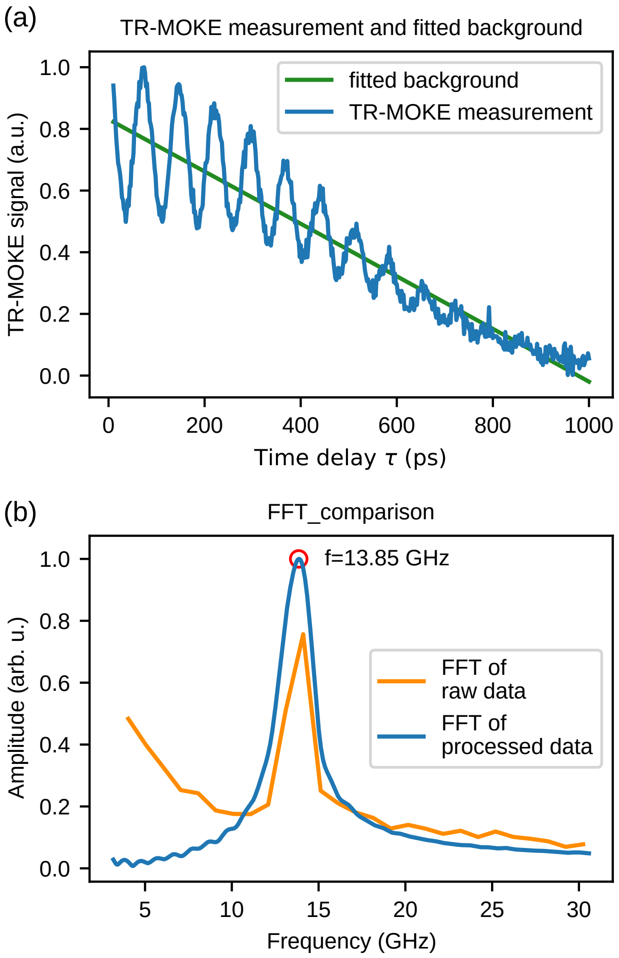

Figure 1. (a) The measured TR-MOKE signal (a.u.) in dependence on the delay time (ps) presented as a blue line. In order to improve postprocessing – getting the exact value of fundamental mode frequency, the background of the TR-MOKE signal was fitted, according to Eq.1 - presented by a green line. (b) Result of Fast Fourier Transform done from the measured signal in order to extract the frequency of signal measured. An orange line presents the result of FFT directly calculated from the measured signal. A blue line presents the result of FFT done from the post-processed signal - after removing background, zero-padding, and autocorrelation of the signal.

B. Fundamental mode frequency calculations

The solution of magnetostatic Maxwell equations with electrodynamic boundary conditions allows finding the demagnetizing field $\mathbf{H}_d(\mathbf{r})$ for a given magnetization distribution $\mathbf{M}(\mathbf{r})$: $\mathbf{H}_d(\mathbf{r}) = \int_V \mathbf{G}(\mathbf{r},\mathbf{r}')\mathbf{M}(\mathbf{r}')d\mathbf{r}'$, where $\mathbf{G}(\mathbf{r},\mathbf{r}')$ is a tensorial operator (tensorial magnetostatic Green's function), expressing the non-local character of dipolar interactions. For the flat stripe, the ratio of thickness t to width w is small ($q = d/w \ll 1$), the expression for $\mathbf{G}$ can be significantly simplified – there is no dependence along the stripes ($y-$direction), and we can assume a uniform magnetization profile across the stripe's thickness ($z-$direction). These assumptions allow us to consider only the zz and xx components of the $\mathbf{G}$ tensor. The eigenvalue problem for an integral operator (expressed by G_{zz} or G_{xx}) will take a form[1]:

$$\lambda_z m_\alpha(\xi) = \int_{-1/2}^{1/2} G_{\alpha,\alpha}(\xi,\xi')m_\alpha(\xi')d\xi', \tag{2}$$

where $\alpha = \{z,x\}$, $\xi = x/w$, $\xi' = x'/w$ and

$$G_{zz}(\xi,\xi') = \frac{1}{q}\ln\frac{(\xi-\xi')^4}{(q^2+(\xi-\xi')^2)^2}. \tag{3}$$

The G_{xx} and G_{zz} are related to each other: $G_{xx}(\xi,\xi') + G_{zz}(\xi,\xi') = -4\pi\delta(\xi,\xi')$. The eigenfunctions of the eigenvalue problems (2) are magnetization profiles for the modes quantized in the stripes, in $x-$direction: $m_\alpha(\xi)$. The corresponding eigenvalues λ_α allow us to determine the components of dynamic demagnetizing fields $h_{d,\alpha} = -\frac{1}{4\pi}\lambda_\alpha$. Note that: $\lambda_x + \lambda_z = -\frac{1}{4\pi}$.

In the periodic sequence of stripes, the magnetization profiles are phase-shifted by the factor e^{inka}, where n is the index numbering the successive stripes, k is the wave vector, and $a = g + w$ is the period for the structure. The spin wave profile in the nth stripe $m_{\alpha,n}(x) := m_\alpha(x+na)$ can be expressed in terms of the periodic factor of the Bloch function $\tilde{m}_\alpha(x) = \tilde{m}_\alpha(x+na)$:

$$m_{\alpha,n}(\xi) = \tilde{m}_\alpha(\xi)e^{ikw(\xi+na/w)}, \tag{4}$$

which can be determined from the following integral eigenvalue problem[2]:

$$\lambda_\alpha \tilde{m}_\alpha(\xi) = \sum_{n=-\infty}^{\infty} \int_{-1/2}^{1/2} G_{\alpha,\alpha}(\xi,\xi'+na/w)\tilde{m}_\alpha(\xi')e^{ikw(\xi'-\xi+na/w)}d\xi', \tag{5}$$

where $G_{\alpha,\alpha}$ is zz or xx component of magnetostatic Green's function for a single stripe.

We were looking for the fundamental mode in the array of stripes. Therefore, we solved the integral eigenvalue problem (5) for $k = 0$ and looked for its lowest eigenfrequency. Under such conditions, the profiles $m_{n,\alpha}$ do not have modes inside the stripes and have the same constant phase for each stripe. We solved the eigenproblem (5) for a finite number of the elements (stripes) in the sum ($n = -10,\ldots,10$) and for the zz component of the tensor $\mathbf{G}$. The integral was approximated by the sum, according to the Nyström method, and the integral eigenvalue problem was converted to an algebraic one, solved numerically. The lowest eigenvalue λ_z was used to determine the demagnetizing factors for the fundamental node: $N_z = -\frac{1}{4\pi}\lambda_z$, $N_x = 1 - N_z$, $N_y = 0$ in the array of stripes. The numerically calculated demagnetizing factors were inserted into the Kittel equation, i.e., the equation for the fundamental mode frequency[1] (Eq.6 in the manuscript).

C. Equilibrium position of static magnetization

The equilibrium orientation of magnetization, equivalent to the zeroing of the torque in Landau-Lifshitz equation, can be calculated as a minimum of free energy density $F(\theta,\theta_M)$[3], where θ and θ_M are the angles between the normal to the plane of stripes ($z-$direction) and: the external field H_0 and static magnetization, respectively:

$$F(\theta,\theta_M) = -\mu_0 M_S H_0 \cos(\theta-\theta_M) + \frac{1}{2}\mu_0 M_S^2 N_z \cos^2\theta. \tag{6}$$

We assumed that the external field is applied in the $z-y$ plane. The equilibrium position of magnetization $\theta_{M,0}$ can be found as extremum (minimum) of free energy density $\frac{\partial}{\partial\theta_M}F(\theta,\theta_M) = 0$, which leads to the following equation:

$$H_0 \sin(\theta_{M,0}-\theta) - \frac{1}{2}M_S N_z \sin(2\theta_{M,0}) = 0. \tag{7}$$

For the considered system, where $\theta = 60^\circ$, $\mu_0 M_S = 1690$ mT $\gg \mu_0 H_0 = 150$ mT, and the stripes are flat (N_z ranging from 0.94 to 1, depending on the widths of stripes w and separations between them g), we obtain the value $\theta_{M,0} \approx 2.5^\circ$. This allowed us to assume that the magnetization is oriented in-plane.

References

1. Guslienko, K. Y., Demokritov, S., Hillebrands, B. & Slavin, A. Effective dipolar boundary conditions for dynamic magnetization in thin magnetic stripes. *Phys. Rev. B* **66**, 132402, DOI: 10.1103/PhysRevB.66.132402 (2002).

2. Kostylev, M. P., Stashkevich, A. A. & Sergeeva, N. A. Collective magnetostatic modes on a one-dimensional array of ferromagnetic stripes. *Phys. Rev. B* **69**, 064408, DOI: 10.1103/PhysRevB.69.064408 (2004).

3. Vonsovskii, S. V. *Ferromagnetic Resonance* (Pergamon, 1966).
